# Efficacy and safety of TDF/FTC-containing, first-line HAART in clinical practice: 3-year data from the German outpatient cohort

**DOI:** 10.1186/1758-2652-13-S4-P12

**Published:** 2010-11-08

**Authors:** J van Lunzen, G Fätkenheuer, T Lutz, S Klauke, S Mauss, H Knechten, P Braun, L Gallo, J Goldbach

**Affiliations:** 1 Universitätsklinikum Hamburg-Eppendorf, Ambulanzzentrum des UKE GmbH, Hamburg, Germany; 2 Med. Einrichtungen der Universität Köln, Klinik I für Innere Medizin, Köln, Germany; 3 Infektiologikum Frankfurt, Praxis Friedensstraße, Frankfurt, Germany; 4 Infektiologikum Frankfurt, Praxis Stresemannallee, Frankfurt, Germany; 5 Center for HIV and Hepatogastroenterology, Düsseldorf, Germany; 6 Praxiszentrum Blondelstrasse, Aachen, Germany; 7 Gilead Sciences, Martinsried, Germany

## Purpose of the study

First line HAART with tenofovir DF (TDF) and emtricitabine (FTC) in pivotal trials has been associated with high efficacy and good tolerability. However, real-life clinical practice often differs from clinical trials due to co-morbidities, co-infections, and less intensive clinical monitoring.

## Methods

Between July 2005 and August 2006, 534 HIV^+^ antiretroviral naïve patients (pts) from 50 German centres enrolled in this non-interventional cohort. All patients were to be followed for three years every three months to monitor and document efficacy (VL, CD4), renal safety, tolerability, regimen changes and resistance profile. All patients received TDF+FTC as a single tablet fixed-combination (Truvada, TVD) mostly in combination with either NNRTI or PI/r as their first regimen.

## Summary of results

As of April 2010, three years of therapy have been documented for 330/534 (61.8%) pts; 81% male; median age was 39 years. Clinical AIDS diagnosis was documented in 22% pts; 46% pts started therapy with median 211 (IQR: 111-297) CD4 cells/mm^3^. TVD was combined with NNRTI (efavirenz (EFV)27%, nevirapine 16%), PI/r (54%) or other (3%). In an as treated analysis after 36 months, 91% of pts achieved VL<50 copies/mL (VL<200 copies/mL: 97.1%; VL<500 copies/ml: 99%). Median CD4 cell count increased to 472 cells/mm^3^ (IQR: 341-631). Regimen with TVD showed a good safety profile and 36 pts were switched to a single tablet regimen with EFV/TDF/FTC (Atripla); 113 adverse events (AEs) of any grade were reported in 73/534 pts (13.7%); 15 of these were rated serious. 21 (3.9%) pts discontinued the TVD regimen due to AEs. Most of them (n=13) discontinued within the first six months. Renal abnormalities of any grade were reported in 10 pts (8.8% of all AEs). Median creatinine clearance was 109.0 mL/min (n=444) at baseline and 103.3 mL/min (n=287) after 36 months. Virological failure as a reason for discontinuation was documented in 12 pts; in 11 failing pts genotyping was performed and detected M184V (n=2) or K65R (n=2) among other NRTI, PI or NNRTI mutations. One failing patient had shown M184V at baseline.

## Conclusions

During three years of follow-up, overall safety of TVD was good. Virological failure was rare (12 pts) and K65R was detected in only 2 failing patients. First line HAART with Truvada (TDF/FTC) plus an NNRTI or PI/r in clinical practice showed comparable efficacy to that observed in controlled clinical trials.

